# 

**Published:** 1895-06

**Authors:** 


### CONTENTS.

ORIGINAL COMMUNICATIONS—

The Practical Treatment of "Cancer" of the Skin. By M. B. Hutchins, M.D., Atlanta, Ga. .... 193

Rudimentary Preparation, Compared with Higher Education, for Specialties in Medicine and Surgery. By J. McFadden Gaston, M.D., Atlanta, Ga. .... 200

The Necessity for Proper Care of School Children's Eyes. By Dunbar Roy, A.B., M.D., Atlanta, Ga .... 207

Treatment of Acute Croupous Pneumonia. By J. S. Cain, M.D., Nashville, Tenn.. 217

Report of Part of My Surgical Work for the Six Months Ending April 15th, with Remarks. By J. B. S. Holmes, M.D., Atlanta, Ga. .... 223

SOCIETY REPORTS—

Richmond Academy of Medicine and Surgery .... 238

CORRESPONDENCE—

New York Letter .... 241

EDITORIAL—

The Late Meetings in Baltimore .... 246

The Christian Advocate and the Keeley Cure .... 250

BOOK REVIEWS .... 252

MEDICAL ITEMS .... 255

ADVERTISERS' INDEX TO ADVERTISEMENTS .... xii

MEDICAL ITEMS—Continued .... x, xi

CLASSIFIED INDEX TO ADVERTISEMENTS .... xxxi.

